# Retraction

**DOI:** 10.1002/cam4.5822

**Published:** 2023-04-12

## Abstract

Tong L, Ao Y, Zhang H, Wang K, Wang Y, Ma Q. Long noncoding RNA NORAD is upregulated in epithelial ovarian cancer and its downregulation suppressed cancer cell functions by competing with miR‐155‐5p. *Cancer Med*. 2019;8:4782–4791. https://doi.org/10.1002/cam4.2350

The above article, published online on 28 June 2019 in Wiley Online Library (wileyonlinelibrary.com), has been retracted by agreement between the authors, the journal Editor in Chief, Dr Stephen Tait and John Wiley and Sons Ltd. The retraction has been agreed due to scientific inconsistences invalidating the conclusions presented.

Tong L, Ao Y, Zhang H, Wang K, Wang Y, Ma Q. Long noncoding RNA NORAD is upregulated in epithelial ovarian cancer and its downregulation suppressed cancer cell functions by competing with miR‐155‐5p. *Cancer Med*. 2019;8:4782–4791. https://doi.org/10.1002/cam4.2350

The above article, published online on 28 June 2019 in Wiley Online Library (wileyonlinelibrary.com), has been retracted by agreement between the authors, the journal Editor in Chief, Dr Stephen Tait and John Wiley and Sons Ltd. The retraction has been agreed due to scientific inconsistences invalidating the conclusions presented.

